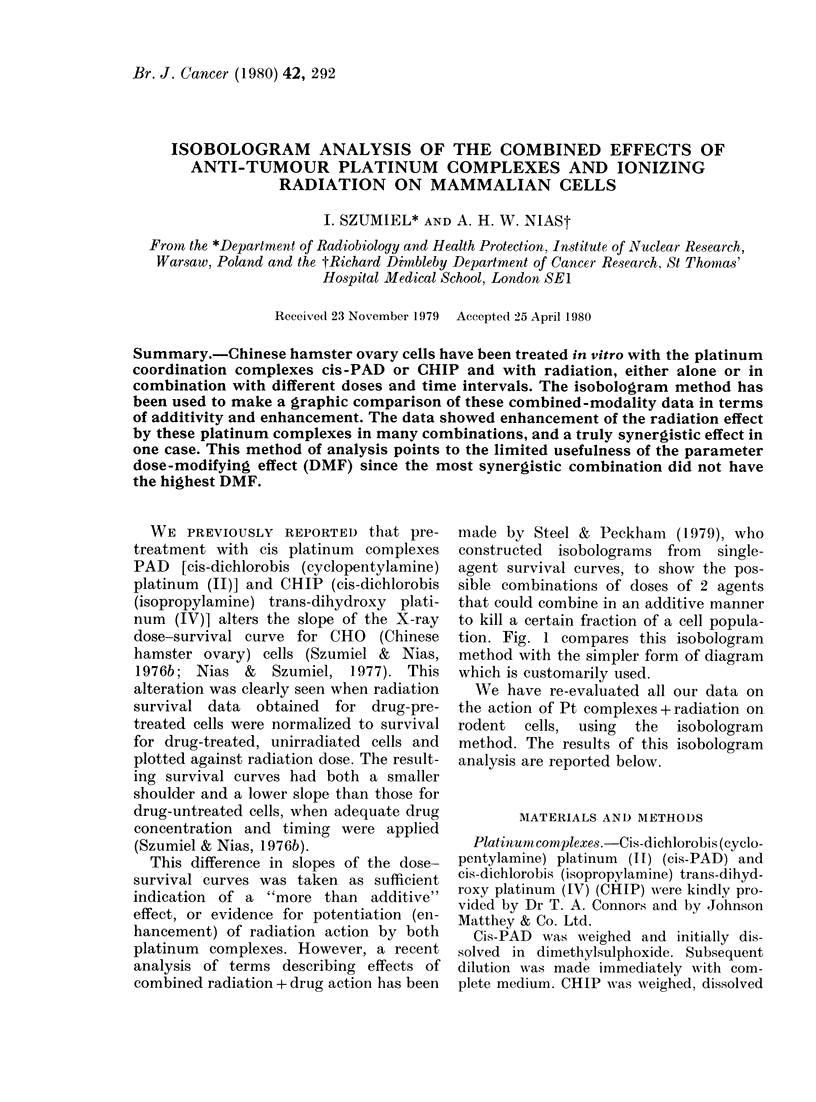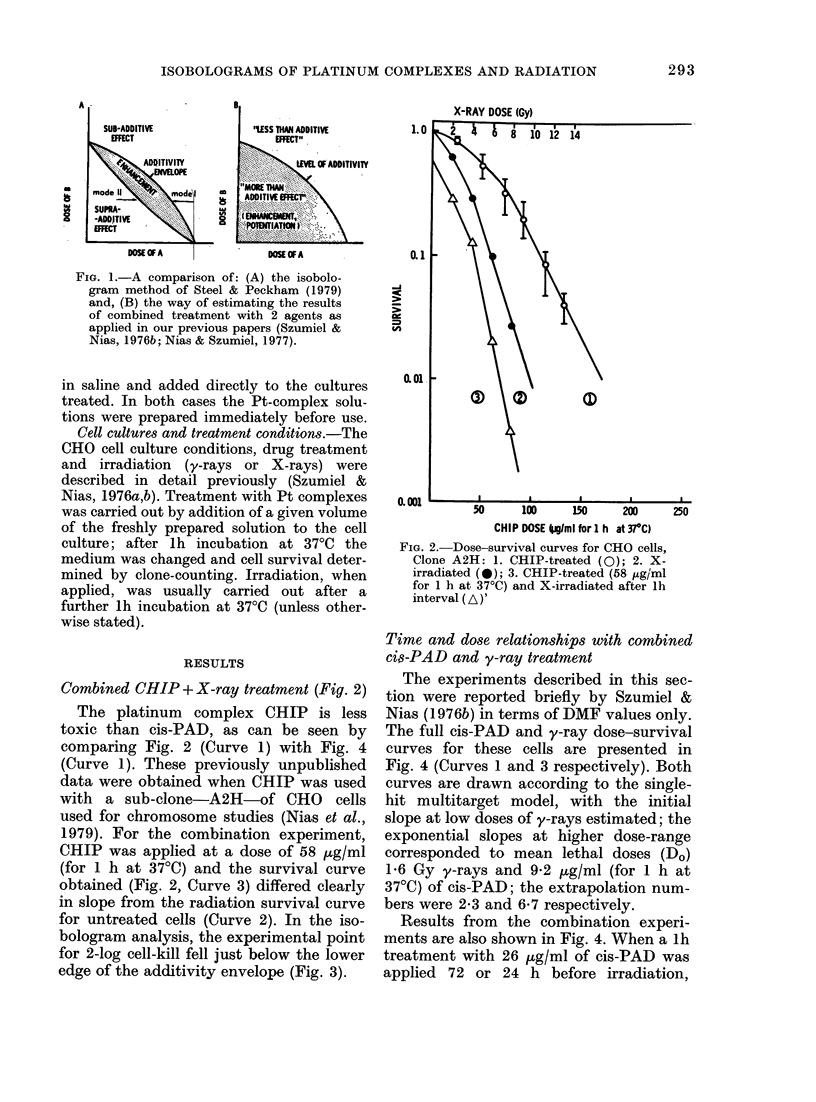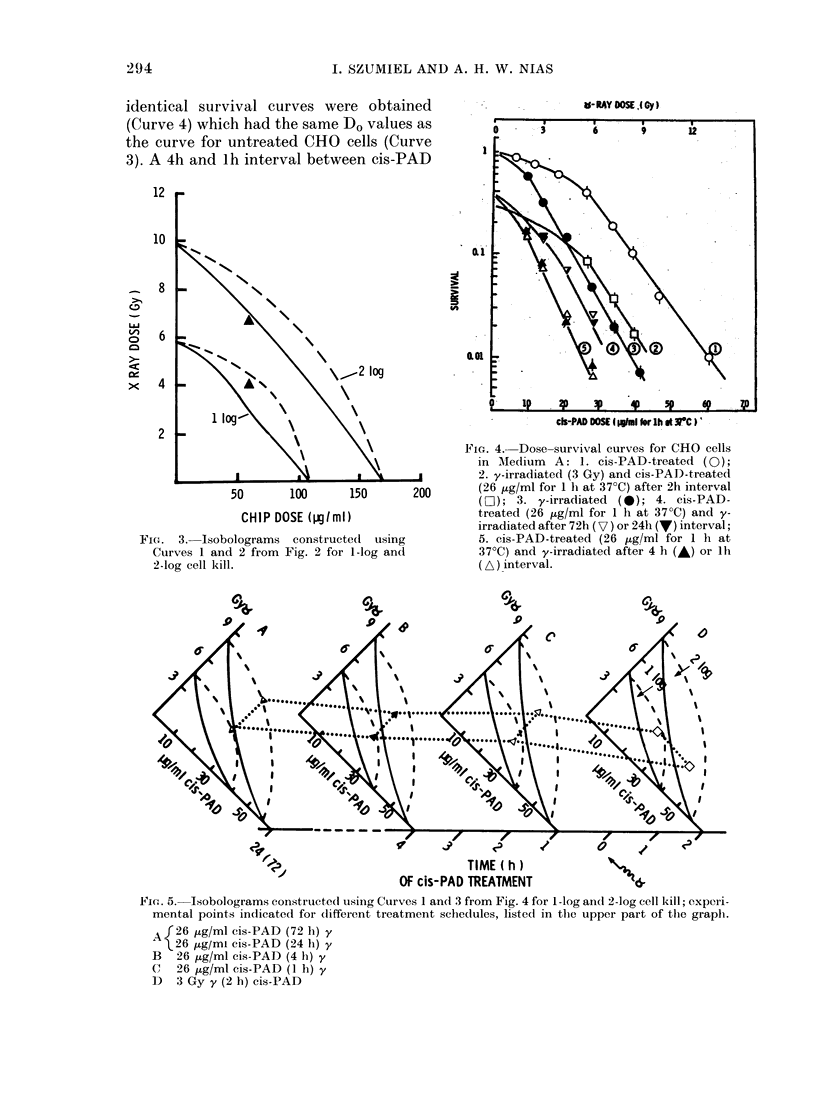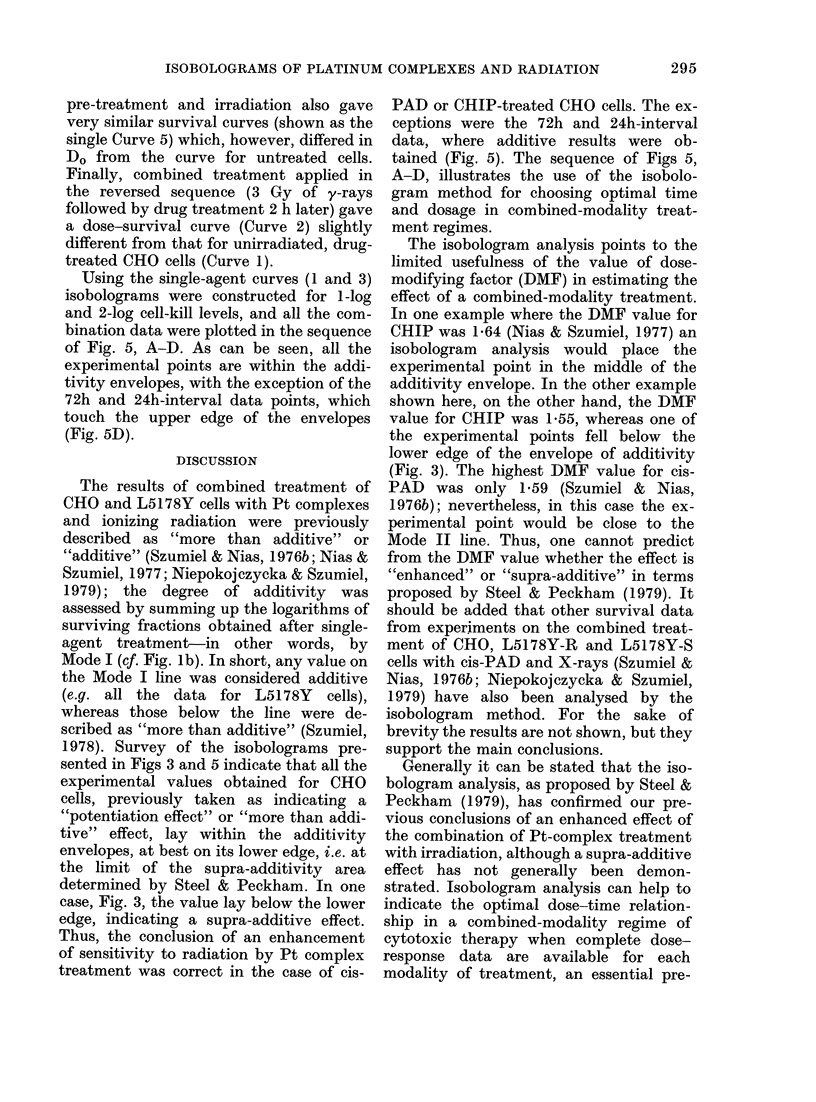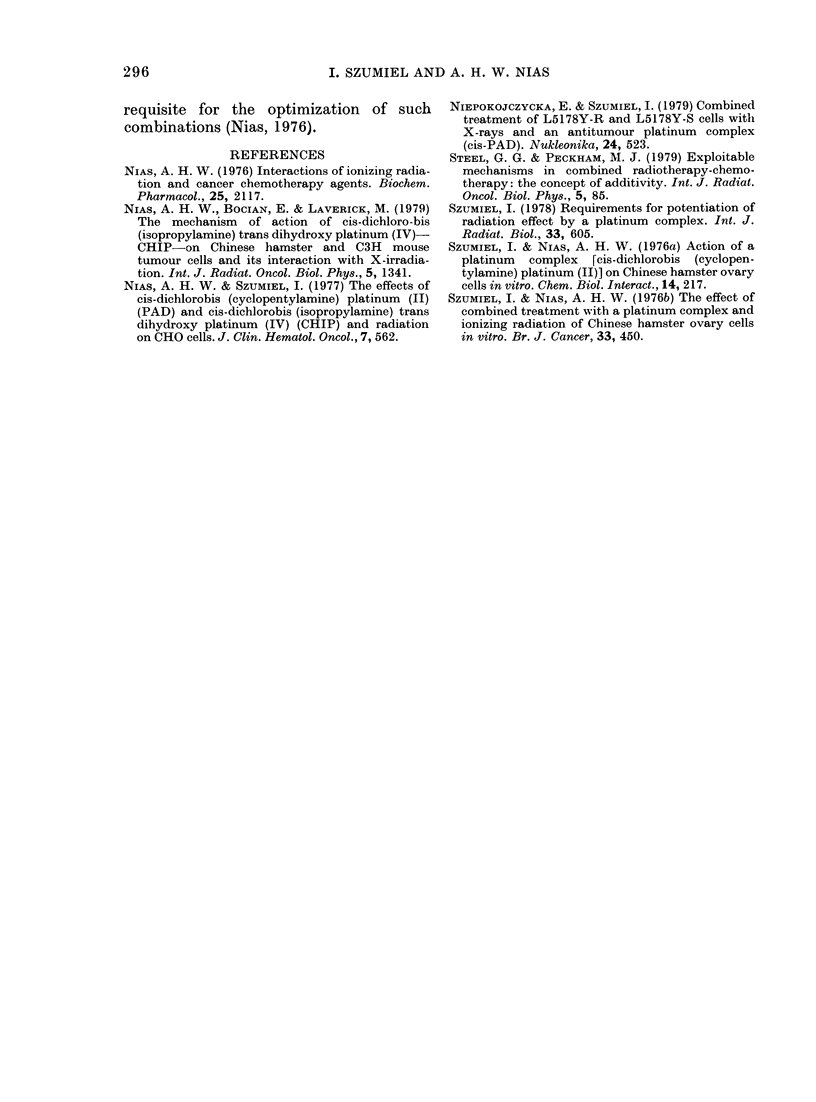# Isobologram analysis of the combined effects of anti-tumour platinum complexes and ionizing radiation on mammalian cells.

**DOI:** 10.1038/bjc.1980.229

**Published:** 1980-08

**Authors:** I. Szumiel, A. H. Nias

## Abstract

Chinese hamster ovary cells have been treated in vitro with the platinum coordination complexes cis-PAD or CHIP and with radiation, either alone or in combination with different doses and time intervals. The isobologram method has been used to make a graphic comparison of these combined-modality data in terms of additivity and enhancement. The data showed enhancement of the radiation effect by these platinum complexes in many combinations, and a truly synergistic effect in one case. This method of analysis points to the limited usefulness of the parameter dose-modifying effect (DMF) since the most synergistic combination did not have the highest DMF.


Br. J. Cancer (1980) 42, 292

### ISOBOLOGRAM ANALYSIS OF THE COMBINED EFFECTS OF ANTI-TUMOUR PLATINUM COMPLEXES AND IONIZING RADIATION ON MAMMALIAN CELLS

I. SZUMIEL* AND A. H. W. NIAS†

*From the *Department of Radiobiology and Health Protection, Institute of Nuclear Research, Warsaw, Poland and the †Richard Dimbleby Department of Cancer Research, St Thomas' Hospital Medical School, London SE1*

Received 23 November 1979 Accepted 25 April 1980

**Summary.—Chinese hamster ovary cells have been treated *in vitro* with the platinum coordination complexes cis-PAD or CHIP and with radiation, either alone or in combination with different doses and time intervals. The isobologram method has been used to make a graphic comparison of these combined-modality data in terms of additivity and enhancement. The data showed enhancement of the radiation effect by these platinum complexes in many combinations, and a truly synergistic effect in one case. This method of analysis points to the limited usefulness of the parameter dose-modifying effect (DMF) since the most synergistic combination did not have the highest DMF.**

WE PREVIOUSLY REPORTED that pretreatment with cis platinum complexes PAD [cis-dichlorobis (cyclopentylamine) platinum (II)] and CHIP (cis-dichlorobis (isopropylamine) trans-dihydroxy platinum (IV)] alters the slope of the X-ray dose–survival curve for CHO (Chinese hamster ovary) cells (Szumiel & Nias, 1976*b*; Nias & Szumiel, 1977). This alteration was clearly seen when radiation survival data obtained for drug-pretreated cells were normalized to survival for drug-treated, unirradiated cells and plotted against radiation dose. The resulting survival curves had both a smaller shoulder and a lower slope than those for drug-untreated cells, when adequate drug concentration and timing were applied (Szumiel & Nias, 1976*b*).

This difference in slopes of the dose–survival curves was taken as sufficient indication of a "more than additive" effect, or evidence for potentiation (enhancement) of radiation action by both platinum complexes. However, a recent analysis of terms describing effects of combined radiation + drug action has been made by Steel & Peckham (1979), who constructed isobolograms from single-agent survival curves, to show the possible combinations of doses of 2 agents that could combine in an additive manner to kill a certain fraction of a cell population. Fig. 1 compares this isobologram method with the simpler form of diagram which is customarily used.

We have re-evaluated all our data on the action of Pt complexes + radiation on rodent cells, using the isobologram method. The results of this isobologram analysis are reported below.

### MATERIALS AND METHODS

*Platinum complexes.*—Cis-dichlorobis (cyclopentylamine) platinum (II) (cis-PAD) and cis-dichlorobis (isopropylamine) trans-dihydroxy platinum (IV) (CHIP) were kindly provided by Dr T. A. Connors and by Johnson Matthey & Co. Ltd.

Cis-PAD was weighed and initially dissolved in dimethylsulphoxide. Subsequent dilution was made immediately with complete medium. CHIP was weighed, dissolved

FIG. 1.—A comparison of: (A) the isobologram method of Steel & Peckham (1979) and, (B) the way of estimating the results of combined treatment with 2 agents as applied in our previous papers (Szumiel & Nias, 1976*b*; Nias & Szumiel, 1977).

in saline and added directly to the cultures treated. In both cases the Pt-complex solutions were prepared immediately before use.

*Cell cultures and treatment conditions.*—The CHO cell culture conditions, drug treatment and irradiation ($\gamma$-rays or X-rays) were described in detail previously (Szumiel & Nias, 1976*a*,*b*). Treatment with Pt complexes was carried out by addition of a given volume of the freshly prepared solution to the cell culture; after 1h incubation at 37°C the medium was changed and cell survival determined by clone-counting. Irradiation, when applied, was usually carried out after a further 1h incubation at 37°C (unless otherwise stated).

### RESULTS

*Combined CHIP + X-ray treatment* (*Fig.* 2)

The platinum complex CHIP is less toxic than cis-PAD, as can be seen by comparing Fig. 2 (Curve 1) with Fig. 4 (Curve 1). These previously unpublished data were obtained when CHIP was used with a sub-clone—A2H—of CHO cells used for chromosome studies (Nias *et al.*, 1979). For the combination experiment, CHIP was applied at a dose of 58 $\mu$g/ml (for 1 h at 37°C) and the survival curve obtained (Fig. 2, Curve 3) differed clearly in slope from the radiation survival curve for untreated cells (Curve 2). In the isobologram analysis, the experimental point for 2-log cell-kill fell just below the lower edge of the additivity envelope (Fig. 3).

FIG. 2.—Dose–survival curves for CHO cells, Clone A2H: 1. CHIP-treated (○); 2. X-irradiated (●); 3. CHIP-treated (58 $\mu$g/ml for 1 h at 37°C) and X-irradiated after 1h interval (△)'

*Time and dose relationships with combined cis-PAD and $\gamma$-ray treatment*

The experiments described in this section were reported briefly by Szumiel & Nias (1976*b*) in terms of DMF values only. The full cis-PAD and $\gamma$-ray dose–survival curves for these cells are presented in Fig. 4 (Curves 1 and 3 respectively). Both curves are drawn according to the single-hit multitarget model, with the initial slope at low doses of $\gamma$-rays estimated; the exponential slopes at higher dose-range corresponded to mean lethal doses ($D_0$) 1·6 Gy $\gamma$-rays and 9·2 $\mu$g/ml (for 1 h at 37°C) of cis-PAD; the extrapolation numbers were 2·3 and 6·7 respectively.

Results from the combination experiments are also shown in Fig. 4. When a 1h treatment with 26 $\mu$g/ml of cis-PAD was applied 72 or 24 h before irradiation, identical survival curves were obtained (Curve 4) which had the same $D_0$ values as the curve for untreated CHO cells (Curve 3). A 4h and 1h interval between cis-PAD

FIG. 3.—Isobolograms constructed using Curves 1 and 2 from Fig. 2 for 1-log and 2-log cell kill.

FIG. 4.—Dose-survival curves for CHO cells in Medium A: 1. cis-PAD-treated (○); 2. γ-irradiated (3 Gy) and cis-PAD-treated (26 µg/ml for 1 h at 37°C) after 2h interval (□); 3. γ-irradiated (●); 4. cis-PAD-treated (26 µg/ml for 1 h at 37°C) and γ-irradiated after 72h (▽) or 24h (▼) interval; 5. cis-PAD-treated (26 µg/ml for 1 h at 37°C) and γ-irradiated after 4 h (▲) or 1h (△) interval.

FIG. 5.—Isobolograms constructed using Curves 1 and 3 from Fig. 4 for 1-log and 2-log cell kill; experimental points indicated for different treatment schedules, listed in the upper part of the graph.

A { 26 µg/ml cis-PAD (72 h) γ

  26 µg/ml cis-PAD (24 h) γ

B 26 µg/ml cis-PAD (4 h) γ

C 26 µg/ml cis-PAD (1 h) γ

D 3 Gy γ (2 h) cis-PAD

pre-treatment and irradiation also gave very similar survival curves (shown as the single Curve 5) which, however, differed in $D_0$ from the curve for untreated cells. Finally, combined treatment applied in the reversed sequence (3 Gy of $\gamma$-rays followed by drug treatment 2 h later) gave a dose–survival curve (Curve 2) slightly different from that for unirradiated, drug-treated CHO cells (Curve 1).

Using the single-agent curves (1 and 3) isobolograms were constructed for 1-log and 2-log cell-kill levels, and all the combination data were plotted in the sequence of Fig. 5, A–D. As can be seen, all the experimental points are within the additivity envelopes, with the exception of the 72h and 24h-interval data points, which touch the upper edge of the envelopes (Fig. 5D).

### DISCUSSION

The results of combined treatment of CHO and L5178Y cells with Pt complexes and ionizing radiation were previously described as "more than additive" or "additive" (Szumiel & Nias, 1976*b*; Nias & Szumiel, 1977; Niepokojczycka & Szumiel, 1979); the degree of additivity was assessed by summing up the logarithms of surviving fractions obtained after single-agent treatment—in other words, by Mode I (*cf.* Fig. 1b). In short, any value on the Mode I line was considered additive (*e.g.* all the data for L5178Y cells), whereas those below the line were described as "more than additive" (Szumiel, 1978). Survey of the isobolograms presented in Figs 3 and 5 indicate that all the experimental values obtained for CHO cells, previously taken as indicating a "potentiation effect" or "more than additive" effect, lay within the additivity envelopes, at best on its lower edge, *i.e.* at the limit of the supra-additivity area determined by Steel & Peckham. In one case, Fig. 3, the value lay below the lower edge, indicating a supra-additive effect. Thus, the conclusion of an enhancement of sensitivity to radiation by Pt complex treatment was correct in the case of cis-PAD or CHIP-treated CHO cells. The exceptions were the 72h and 24h-interval data, where additive results were obtained (Fig. 5). The sequence of Figs 5, A–D, illustrates the use of the isobologram method for choosing optimal time and dosage in combined-modality treatment regimes.

The isobologram analysis points to the limited usefulness of the value of dose-modifying factor (DMF) in estimating the effect of a combined-modality treatment. In one example where the DMF value for CHIP was 1·64 (Nias & Szumiel, 1977) an isobologram analysis would place the experimental point in the middle of the additivity envelope. In the other example shown here, on the other hand, the DMF value for CHIP was 1·55, whereas one of the experimental points fell below the lower edge of the envelope of additivity (Fig. 3). The highest DMF value for cis-PAD was only 1·59 (Szumiel & Nias, 1976*b*); nevertheless, in this case the experimental point would be close to the Mode II line. Thus, one cannot predict from the DMF value whether the effect is "enhanced" or "supra-additive" in terms proposed by Steel & Peckham (1979). It should be added that other survival data from experiments on the combined treatment of CHO, L5178Y-R and L5178Y-S cells with cis-PAD and X-rays (Szumiel & Nias, 1976*b*; Niepokojczycka & Szumiel, 1979) have also been analysed by the isobologram method. For the sake of brevity the results are not shown, but they support the main conclusions.

Generally it can be stated that the isobologram analysis, as proposed by Steel & Peckham (1979), has confirmed our previous conclusions of an enhanced effect of the combination of Pt-complex treatment with irradiation, although a supra-additive effect has not generally been demonstrated. Isobologram analysis can help to indicate the optimal dose–time relationship in a combined-modality regime of cytotoxic therapy when complete dose–response data are available for each modality of treatment, an essential prerequisite for the optimization of such combinations (Nias, 1976).

### REFERENCES

NIAS, A. H. W. (1976) Interactions of ionizing radiation and cancer chemotherapy agents. *Biochem. Pharmacol.*, **25**, 2117.

NIAS, A. H. W., BOCIAN, E. & LAVERICK, M. (1979) The mechanism of action of cis-dichloro-bis (isopropylamine) trans dihydroxy platinum (IV)—CHIP—on Chinese hamster and C3H mouse tumour cells and its interaction with X-irradiation. *Int. J. Radiat. Oncol. Biol. Phys.*, **5**, 1341.

NIAS, A. H. W. & SZUMIEL, I. (1977) The effects of cis-dichlorobis (cyclopentylamine) platinum (II) (PAD) and cis-dichlorobis (isopropylamine) trans dihydroxy platinum (IV) (CHIP) and radiation on CHO cells. *J. Clin. Hematol. Oncol.*, **7**, 562.

NIEPOKOJCZYCKA, E. & SZUMIEL, I. (1979) Combined treatment of L5178Y-R and L5178Y-S cells with X-rays and an antitumour platinum complex (cis-PAD). *Nukleonika*, **24**, 523.

STEEL, G. G. & PECKHAM, M. J. (1979) Exploitable mechanisms in combined radiotherapy-chemotherapy: the concept of additivity. *Int. J. Radiat. Oncol. Biol. Phys.*, **5**, 85.

SZUMIEL, I. (1978) Requirements for potentiation of radiation effect by a platinum complex. *Int. J. Radiat. Biol.*, **33**, 605.

SZUMIEL, I. & NIAS, A. H. W. (1976*a*) Action of a platinum complex [cis-dichlorobis (cyclopentylamine) platinum (II)] on Chinese hamster ovary cells *in vitro*. *Chem. Biol. Interact.*, **14**, 217.

SZUMIEL, I. & NIAS, A. H. W. (1976*b*) The effect of combined treatment with a platinum complex and ionizing radiation of Chinese hamster ovary cells *in vitro*. *Br. J. Cancer*, **33**, 450.